# High Performance Field-Effect Transistors Based on Partially Suspended 2D Materials via Block Copolymer Lithography

**DOI:** 10.3390/polym13040566

**Published:** 2021-02-14

**Authors:** Simon Kim, Su Eon Lee, Jun Hyun Park, Jin Yong Shin, Bom Lee, Heo Yeon Lim, Young Taek Oh, Jun Pyo Hwang, Seung Won Seon, Seung Hee Kim, Tae Sang Yu, Bong Hoon Kim

**Affiliations:** 1 Department of Organic Materials and Fiber Engineering, Soongsil University, 369 Sangdo-ro, Dongjak-gu, Seoul 06978, Korea; kimsimon@soongsil.ac.kr (S.K.); azt0098@gmail.com (J.H.P.); thankusin@gmail.com (J.Y.S.); lb960@naver.com (B.L.); limkitty1004@naver.com (H.Y.L.); hjunpyo1540@soongsil.ac.kr (J.P.H.); ssun@soongsil.ac.kr (S.W.S.); hee17@soongsil.ac.kr (S.H.K.); yts3185@soongsil.ac.kr (T.S.Y.); 2 Department of Smart Wearable Engineering, Soongsil University, 369 Sangdo-ro, Dongjak-gu, Seoul 06978, Korea; gngnv123@soongsil.ac.kr (S.E.L.); 1102071003@soongsil.ac.kr (Y.T.O.)

**Keywords:** block copolymer lithography, nanopatterning, two-dimensional (2D) materials, substrate effect, suspended structure

## Abstract

Although various two-dimensional (2D) materials hold great promise in next generation electronic devices, there are many challenges to overcome to be used in practical applications. One of them is the substrate effect, which directly affects the device performance. The large interfacial area and interaction between 2D materials and substrate significantly deteriorate the device performance. Several top-down approaches have been suggested to solve the problem. Unfortunately, however, they have some drawbacks such as a complicated fabrication process, a high production cost, or a poor mechanical property. Here, we suggest the partially suspended 2D materials-based field-effect transistors (FETs) by introducing block copolymer (BCP) lithography to fabricate the substrate effect-free 2D electronic devices. A wide range of nanometer size holes (diameter = 31~43 nm) is successfully realized with a BCP self-assembly nanopatterning process. With this approach, the interaction mechanism between active 2D materials and substrate is elucidated by precisely measuring the device performance at varied feature size. Our strategy can be widely applied to fabricate 2D materials-based high performance electronic, optoelectronic, and energy devices using a versatile self-assembly nanopatterning process.

## 1. Introduction

Various two-dimensional (2D) materials, such as graphene, semiconductor transition metal dichalcogenides (TMDs), black phosphorus (BP), insulating hexagonal boron nitride (h-BN), and 2D carbides and nitrides (MXenes) have attracted great interest due to their atomically thin structures and versatile electrical and optoelectronic properties such as high flexibility/transparency and outstanding carrier mobility [1,2,3,4,5]. In particular, many studies attempted to replace conventional silicon semiconductor devices by using atomically layered 2D materials, especially for flexible and stretchable electronics [6,7,8,9]. Despite these many advantages of 2D materials as an active layer of electronic devices, they inherently have a huge disadvantage of being extremely sensitive to the surrounding environment, e.g., substrates, due to their single-atom-thickness [10,11,12,13]. The charged impurities resulting from surface roughness significantly deteriorate the performance of 2D materials-based electronic devices and become major obstacles for its practical application [14]. To solve the problem induced by the interaction between 2D materials and substrate, several studies have focused on devising methodologies to suspend 2D materials in order to improve device performance towards intrinsic transport properties [15,16,17,18,19,20,21,22,23]. Usually, they have used micro-scale trench structures fabricated via a conventional top-down microfabrication process [24,25]. However, these approaches have produced other problems, such as a complicated and expensive fabrication process, a poor mechanical property, and especially a low mass-production yield. In this aspect, a bottom-up nanofabrication process based on self-assembly can be a great alternative to fabricate the substrate effect-free 2D electronic devices.

Here, we demonstrate graphene field-effect transistors (G-FETs) that are partially suspended on a nanoporous silicon substrate using self-assembly of a polystyrene-*block*-poly(methylmethacrylate) (PS-*b*-PMMA) diblock copolymer thin film. Block copolymer (BCP) lithography is an emerging self-assembly technique for the large-area patterning of surfaces with regular nanosized features [26,27,28,29]. Unlike the conventional approach where the entire channel area of 2D material-based field-effect transistors (FETs) is suspended, our strategy allows a partial suspension of their channel with a uniform structure and high density. Through this method, we prevent the collapse or degradation of suspended graphene, and obtain excellent structure stability in terms of mechanical properties. The G-FETs with superior performance than conventional graphene transistors are successfully fabricated by minimizing a contact area between graphene and substrate which decreases extrinsic scattering by surface phonons at the SiO_2_ substrate, which directly contacts with graphene. In this study, we fabricate nanopatterned substrates with various feature sizes by controlling the etching process and investigate the mechanism of substrate effect on the device performance. As a result, in the nanopatterned substrate with decreased substrate effect, we confirm a maximum of 3.2 times higher field-effect mobility (*μ*) by measuring the electrical properties of graphene devices.

## 2. Materials and Methods

### 2.1. Materials

PS-*b*-PMMA diblock copolymer (M_n_ = 205,000 g/mol, Polymer Source INC) and poly(styrene-*ran*-methyl methacrylate) (PS-*r*-PMMA) random copolymer brush (M_n_ = 6400 g/mol, Polymer Source INC) were used to fabricate a BCP nanotemplate on silicon oxide (thickness ~90 nm) wafer (highly p-doped). For 2D materials-based device fabrication, graphene was mechanically exfoliated from highly oriented pyrolytic graphite (HOPG, SPI supplies, Grade SPI-2) [30].

### 2.2. Fabrication of Paritally Suspended G-FETs

#### 2.2.1. Pre-Treatment of Substrate

**Figure 1:**
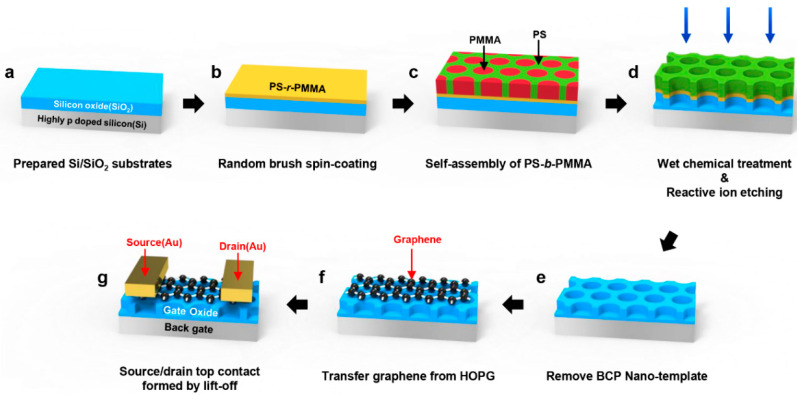
Schematic illustration for the fabrication of partially suspended graphene field-effect transistors (G-FETs) process: (**a**) A silicon oxide wafer is cleaned in sequence with acetone, isopropyl alcohol, and deionized water by sonication. (**b**) A poly(styrene-*ran*-methyl methacrylate) (PS-*r*-PMMA) thin film is spin-coated on a silicon oxide substrate and thermally annealed. (**c**) After washing of the un-grafted PS-*r*-PMMA brush with toluene, a block copolymer (BCP) thin film is spin-coated from solution of the polystyrene-*block*-poly(methylmethacrylate) (PS-*b*-PMMA) diblock copolymer in toluene on a silicon oxide substrate and thermally annealed. (**d**) The BCP nanotemplate is treated in sequence with ultraviolet light, acetic acid, and reactive ion etching process to fabricate a nanopore array on silicon oxide substrate. (**e**) The remaining PS block was removed by sonication in toluene for 60 min by 3 times. (**f**) Graphene is transferred onto nanopatterned silicon oxide substrate. (**g**) Source/drain electrodes are formed with Cr/Au layers.

A silicon oxide wafer was cleaned with acetone, isopropyl alcohol (IPA), and deionized (DI) water with 20 min sonication for each washing step. Subsequently, silicon oxide wafer was sufficiently oxygen plasma treated in vacuum chamber. This process removed most of the impurities on the substrate and provided a highly dense hydroxyl group (–OH) on silicon oxide wafer (Figure 1a) [31,32].

#### 2.2.2. BCP Self-Assembly Process

The surface of silicon oxide wafer was chemically modified by a PS-*r*-PMMA random copolymer brush to provide identical interfacial tensions for the PS and PMMA blocks [33,34]. The PS-*r*-PMMA brush layer (thickness ~40 nm) was deposited by spin-coating process (1 wt% in toluene, 1000 rpm, 60 s) and subsequent thermally annealed at 160 °C for 48 hrs in a vacuum chamber (Figure 1b) [35]. Then, the un-grafted random brush layer was removed by sonication in toluene for 15 min by 3 times. After surface modification, a symmetric diblock copolymer, PS-*b*-PMMA thin film (thickness ~120 nm) was spin-casted (2 wt% in toluene, 1600 rpm, 60 s). Afterwards, thermal annealing was conducted at 220 °C for 48 hrs in a vacuum chamber to produce the surface perpendicular BCP nanodomain (Figure 1c).

#### 2.2.3. SiO_2_ Layer Etching with BCP Nanotemplates

Exposure of the BCP thin film to UV radiation (365 nm, dose of 7.2 J/cm^2^) degraded the PMMA block and induced cross-linking of the PS block [36]. Afterwards, rinsing with dilute acetic acid led to a nanoporous BCP thin film in direct contact with the substrate [37]. For complete removal of PMMA residues, O_2_/Ar gas mixture plasma (source power = 150 W, 5 s) was applied (Figure 1d). During plasma treatment, working pressure and gas flow rate was kept at 92 mTorr and 5/50 sccm (O_2_/Ar), respectively. After PMMA block was removed, silicon oxide layer was etched with CF_4_ gas (source power = 60 W, 30 s, 120 mTorr, 20 sccm) plasma condition [38]. Lastly, the remaining PS block was removed by sonication in toluene for 60 min by 3 times (Figure 1e).

#### 2.2.4. Fabrication of Bottom-Contact G-FETs

The nanopatterned silicon oxide wafer was washed with acetone, IPA, and DI water by sonication for 20 min, sequentially. Monolayer graphene was prepared by mechanically exfoliated HOPG and transferred on the nanopatterned silicon oxide wafer via conventional scotch tape method (Figure 1f) [30]. A thickness of graphene was measured by optical imaging contrast of flakes, and further confirmed by Raman spectroscopy and atomic force microscopy (AFM). Transferred graphene was used to fabricate bottom-contact G-FETs with channel length/width = 2/1 µm using a conventional microfabrication process (Figure 1g) [39].

### 2.3. Characterization

#### 2.3.1. Surface Morphology of Nanopatterned Silicon Oxide Layer

The nanostructure of silicon oxide layer was characterized by field-emission scanning electron microscopy (FE-SEM, Gemini SEM 300, ZEISS, Jena, Germany) and transmission electron microscopy (TEM, Tecnai F20 G2, FEI INC, Hillsboro, OR, USA), respectively.

#### 2.3.2. Raman Spectroscopy of Graphene

Graphene on a flat and nanopatterned silicon oxide thin film was characterized by Raman spectroscope (2GTE70, RENISHAW INC, Wotton-under-Edge, UK) using 512 nm laser.

#### 2.3.3. Semiconductor Characterization System

Transfer characteristics of G-FETs was measured by Keithley 4200-SCS parameter analyzer with a probe station (ST-500-1-4CX, JANIS) under atmosphere condition. The highly boron doped silicon oxide wafer was used as bottom-gate contact of G-FETs. Transfer characteristics was measured with gate voltage (V_GS_) in the range of −20~+20 V with 0.05 V interval.

## 3. Results and Discussion

### 3.1. Nanopatterning of Substrate Using BCP Self-Assembly

**Figure 2:**
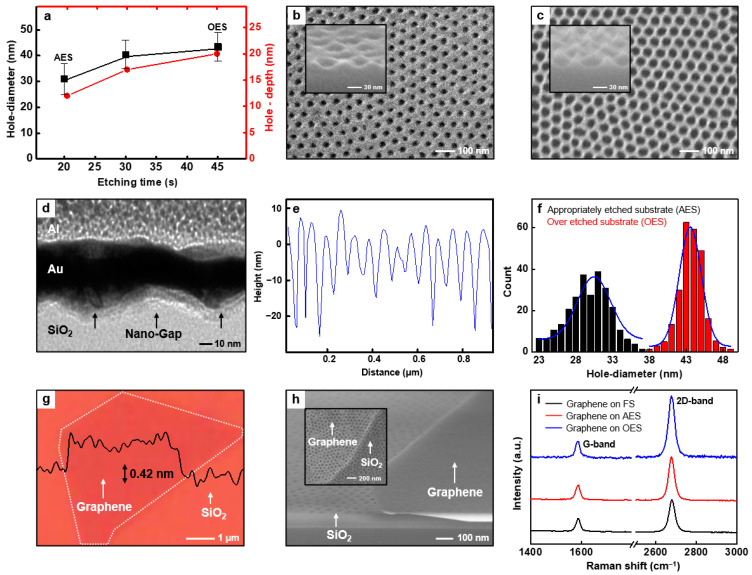
(**a**) A graph for hole diameter/depth vs. etching time. Top view SEM images of (**b**) appropriately etched substrate (AES) and (**c**) over etched substrate (OES). (**d**) A TEM image and (**e**) cross-sectional profile of OES. (**f**) Column chart of hole diameter vs. count of number of holes on the AES and OES in 1 µm^2^ area. (**g**) An OM image and cross-sectional profile of transferred monolayer graphene onto flat silicon oxide substrate (FS, thickness of graphene = 0.42 nm). (**h**) Tilted & top view scanning electron microscope (SEM) images of transferred graphene onto AES during G-FETs fabrication. (**i**) Raman spectra of monolayer graphene on FS, AES, and OES.

The final morphology of the nanopattern on the substrate greatly depends on the etching gas plasma process time. Figure 2a shows the evolution of hole diameter and depth of nanopattern on the substrate at varied CF_4_ gas plasma process time. When CF_4_ gas plasma was applied for 20 s, the appropriately etched substrate (AES) has average hole diameter/depth as 31/12 nm, respectively (Figure 2b). Here AES represents the substrate with same size BCP nanopattern that was used as a dry etching mask and nanopattern transferred to the silicon oxide substrate. This is the case where nanopattern on the silicon oxide substrate have a perfect vertical sidewall. We also fabricate over etched substrate (OES) with minimized contact area between substrate and graphene by increasing CF_4_ gas plasma etching time (45 s). Here, OES is a substrate with larger size of nanopattern transferred to the silicon oxide substrate than the size of BCP nanopattern by increasing dry etching time. In the case of OES, the substrate has a nanopattern with a thinner sidewall than the that of AES. Figure 2c shows the scanning electron microscope (SEM) image of OES with the average hole diameter/depth of 43/20 nm. The transmission electron microscopy (TEM) image and corresponding height cross-sectional profile of OES are shown in Figure 2d,e, respectively. Figure 2f represents the holes diameter size distribution (average diameter of 31/43 nm for AES/OES) of nanopatterns in a 1 µm × 1 µm area (total number of holes = 300) of AES and OES.

### 3.2. Transfer of Graphene to the Substrate and G-FETs Fabrication

To investigate the substrate effect regarding 2D materials-based FETs, we transfer mechanically exfoliated graphene on flat silicon oxide substrate (FS), AES, and OES, respectively. Figure 2g shows optical microscopy (OM) image and a representative cross-sectional profile of monolayer graphene transferred to FS with a thickness of 0.42 nm. Considering the thickness of monolayer graphene (0.34 nm), we conclude that the transfer process of graphene to the substrate has a high technical completion.

Figure 2h shows the top and tilted view SEM images of a graphene transfer printed on AES for G-FETs fabrication. Figure 2i represents the Raman spectra of monolayer graphene transferred to FS, AES, and OES, plotted in black, red, and blue colored lines, respectively. In all samples, no D-band is observed while G-band is observed from FS and AES substrates at 1589 cm^−1^. Interestingly, G-band was left shifted (1585 cm^−1^) at OES, indicating G-band is shifting left as the porosity of substrate increases. While the 2D-band was observed at 2678 cm^−1^ from FS and AES, the band is observed at 2676 cm^−1^ for OES, meaning the band is left shifted as in the case of G-band. In addition, we found that the intensity ratio of 2D-band to G-band (I_2D_/I_G_) is proportional to the porosity of substrate; 1.73, 2.09, and 2.90 for FS, AES, and OES, respectively.

**Figure 3:**
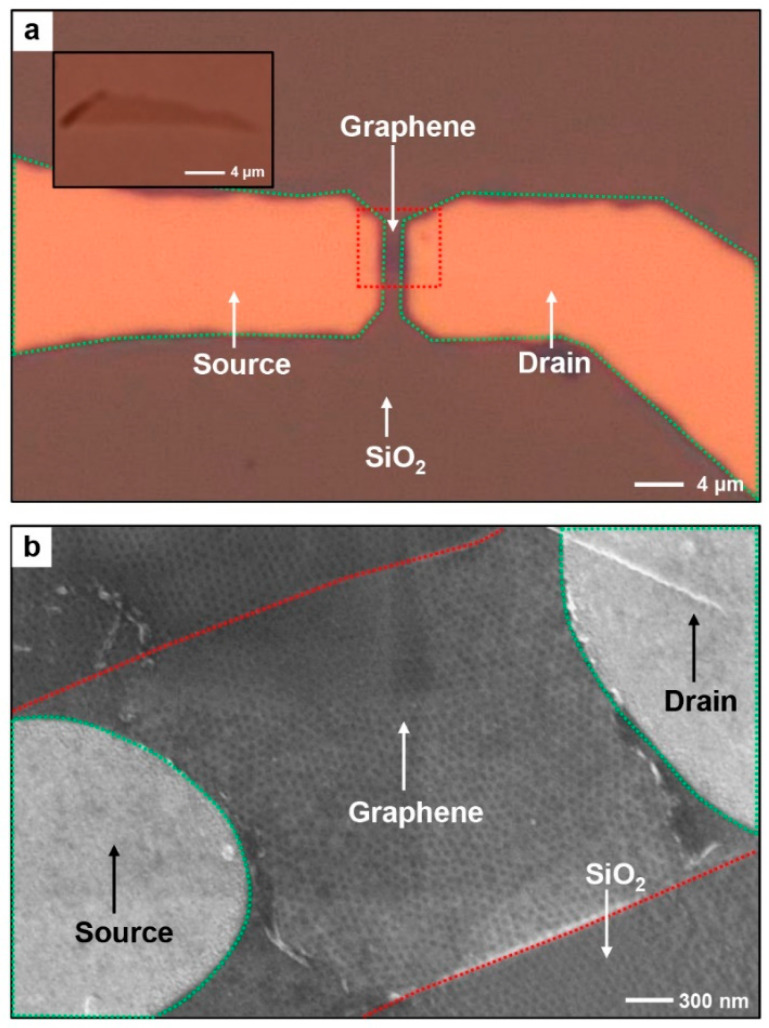
A top view (**a**) OM and (**b**) SEM image of partially suspended G-FETs on AES. Average pore diameter of AES is 31 nm and monolayer graphene is used for the G-FETs fabrication.

Because I_2D_/I_G_ means the degree of doping on the graphene, the increased value indicates that it reaches a more pristine state [40]. When the pore size of BCP nanopattern increases, the contact area of graphene with substrate decreases and it leads to the minimized substrate effect. Therefore, corresponding graphene shows property close to its intrinsic state and its I_2D_/I_G_ is increased. Figure 3a and b show the optical microscopy and top view SEM images of bottom-contact G-FETs on AES, respectively.

### 3.3. Calculation of Capacitance of Insulating Layer

**Figure 4:**
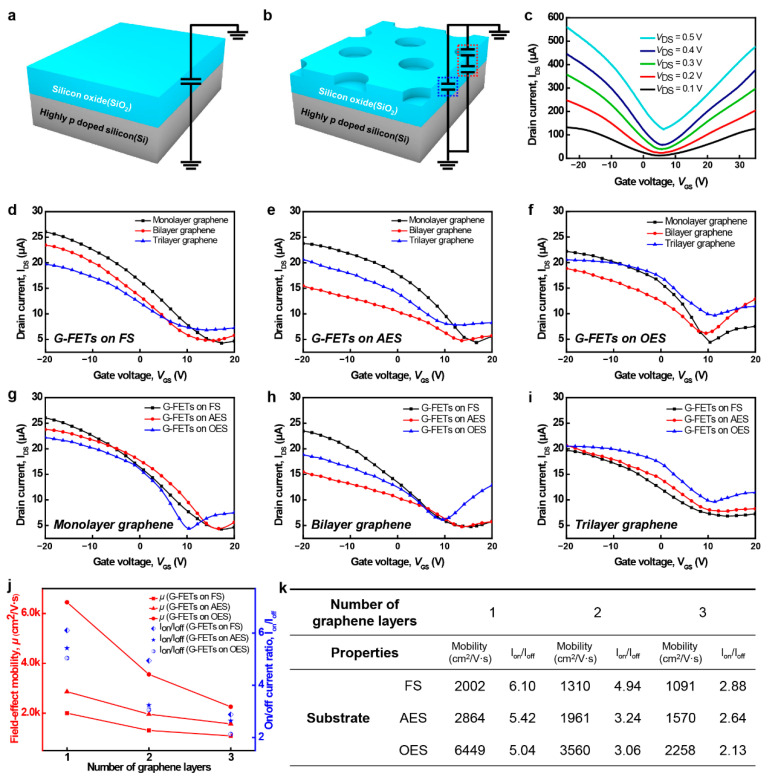
The schematic illustration of the cross-sectional dielectric layers along with their equivalent capacitor circuits for the (**a**) FS and (**b**) nanopatterned substrates (AES and OES). (**c**) Transfer curves of monolayer G-FETs on OES for different values of V_DS_ (V_DS_ = 0.1~0.5 V, internal = 0.1 V). (**d**–**i**) Transfer curves of G-FETs with different number of graphene layers on various substrates. (**j**,**k**) Field-effect mobility (*μ*) and on/off current ratio of G-FETs vs. number of graphene layers on various substrates.

The capacitance of dielectric layer needs to be precisely calculated in order to measure electrical characteristics of G-FETs on nanopatterned substrates. Figure 4a,b show the schematic illustrations and capacitance circuits of dielectric layer from FS and nanopatterned substrates (AES and OES), respectively. In general, the capacitance (*C*) of metal–insulator–metal (MIM) capacitor structure can be expressed as Equation (1):

(1) $$C=\;\varepsilon \;\frac{A}{d}$$

*ε*, *A*, and *d* represent the permittivity of the dielectric material, the surface area of the two plates, and the separation distance between the two plates, respectively. In the case of FS (Figure 4a), it is composed of 90 nm-thick SiO_2_ thin film as a dielectric layer. On the other hand, nanopatterned substrates (AES and OES) have dielectric layers of 90 nm-thick SiO_2_ layer (blue dotted rectangles) and air-SiO_2_ heterostructured layer (red dotted rectangles) connected in parallel (Figure 4b). The thickness of SiO_2_ thin film in the heterostructured layer with suspended graphene on AES and OES are 78 and 70 nm, respectively. The area ratio of hole in this regime is 22% and 43% for AES and OES, respectively. The capacitance of 90 nm-thick SiO_2_ is calculated as 38.7 nF cm^−2^ because the capacitance of the 300 nm-thick SiO_2_ is well-known as 11.6 nF cm^−2^ and the thickness of SiO_2_ and its capacitance are inversely proportional [41]. In the case of nanopatterned substrates (AES and OES), the values of *ε*_air_ = 1.0 and *ε*_SiO__2_ = 3.9 are used to calculate the capacitance of the air. As a result, the capacitances of the 12/78 nm-thick air/SiO_2_ on AES and the 20/70 nm-thick air/SiO_2_ on OES are calculated as 74.4/44.6 nF cm^−2^ and 44.6/49.7 nF cm^−2^, respectively. The capacitance of serially connected capacitors can be calculated as Equation (2):

(2) $$C_{total}=\;\frac{C_{1}C_{2}}{C_{1}+C_{2}\;}$$

On the other hand, the capacitance of parallelly connected capacitors can be obtained from Equation (3):

(3) $$C_{total}=C_{1}+\;C_{2}$$

In the case of air-SiO_2_ heterostructured dielectric layer on AES, the capacitance *C*_air-SiO2_ can be calculated using Equation (2) as 27.9 nF cm^−2^ because 12 nm-thick air and 78 nm-thick SiO_2_ are serially connected. Additionally, we consider a parallelly connected 90 nm-thick SiO_2_ layer of 38.7 nF cm^−2^ and air-SiO_2_ heterostructured dielectric layer of 27.9 nF cm^−2^ in 78:22 ratio, and total capacitance *C*_AES_ was calculated using Equation (3) as *C*_AES_ = 0.78 × *C*_SiO2_ + 0.22 × *C*_air-SiO2_ = 36.3 nF cm^−2^. Likewise, in the case of OES, there are parallel connections with 90 nm-thick SiO_2_ layer of 38.7 nF cm^−2^ and air-SiO_2_ heterostructured dielectric layer of 23.5 nF cm^−2^ in 57:43, and total capacitance *C*_OES_ was calculated as *C*_OES_ = 0.57 × *C*_SiO2_ + 0.43 × *C*_air-SiO2_ = 32.2 nF cm^−2^.

### 3.4. Analysis of G-FETs Electrical Performance

Figure 4c–i show the transfer curves of the G-FETs with different layers of graphenes and types of substrates. Figure 4c represents the transfer characteristics of G-FETs on OES with a single layer graphene at varied drain-source voltage (V_DS_ = 0.1~0.5 V, internal = 0.1 V), indicates that they possess a large working range compatible with diverse power settings (power of device = I_DS_ × V_DS_). Figure 4d–f show the results of I_DS_–V_GS_ curves (V_DS_ = 0.1 V) of G-FETs with different graphene layers on FS (Figure 4d), AES (Figure 4e), and OES (Figure 4f), respectively. From these graphs, we found that the increase of number of graphene layers leads to the raise of the minimum drain current (drain current at the Dirac point) as well as the decrease of on/off current ratio of G-FETs. Figure 4g–i represent the graphs of I_DS_–V_GS_ curves (V_DS_ = 0.1 V) from G-FETs composed of monolayer (Figure 4g), bilayer (Figure 4h), and trilayer (Figure 4i) graphene on different types of substrates. The increase of substrate porosity reduced the contact area between substrate and transferred graphene, which results in the raise of slope in transfer curves as well as the shift of graph towards left. The increase of the slope indicates the increase of dI_DS_/dV_GS_ (transconductance). This is due to the improvement of devices performance by minimizing extrinsic scattering by surface phonons at the SiO_2_ substrate [42]. Also, the left shift of transfer curves at increased substrate porosity represents that G-FETs on OES are more n-doped compared to devices on FS or AES. It is well-known that graphene is slightly p-doped when contacting with silicon oxide layer due to the interfacing effects of the oxygen (O)-terminated substrate [43]. However, in the case of graphene on nanopatterned substrates (AES and OES), the contact area between graphene and substrate is minimized and this is the reason of a graphene with relatively n-doped property compared to a graphene fully contacted with FS. As shown in Figure 4g–i, this phenomenon is universal regardless of number of graphene layers and the effects are getting bigger at fewer graphene layers. Figure 4j shows the comparison of *μ* and bias current on/off ratio (I_on_/I_off_) of G-FETs according to the surface morphology of substrate and number of graphene layers based on transfer curves from Figure 4d–i. The *μ* was calculated using following equation:

(4) $$\mu =\frac{Lg_{m}}{WC_{G}V_{\mathrm{DS}}}$$

Here, *L*, *G*, *W*, and *C*_G_ represents channel length, transconductance, channel width, and gate capacitance of a dielectric layer, respectively. For G, it is calculated from dI_DS_/dV_GS_ of transfer curves of G-FETs and for *C*_G_, the calculated *C*_G_ value at varied substrates were used. In the case of G-FETs composed of monolayer graphene, *μ* with OES increased more than three folds than that of G-FETs on FS (from 2002 cm^2^/V·s to 6449 cm^2^/V·s), and the value of I_on_/I_off_ is decreased from 6.10 to 5.04. Not only monolayer, but also bi- and tri-layer G-FETs show the similar results. Figure 4k summarizes calculated *μ* and I_on_/I_off_ of G-FETs with different layers of graphene and types of substrates.

## 4. Conclusions

The 2D materials were actively used in the next generation electronics/optoelectronics devices [44] due to its promising potential. In some applications, however, there has been innate limitations because such devices performance was significantly deteriorated by interaction between substrate and 2D materials. To overcome this limitation, we engineer the nanostructures of substrate to stably suspend 2D materials and suggested the solution for the performance degradation problems. We demonstrate the partially suspended 2D materials-based FETs by introducing BCP lithography to fabricate the substrate effect-free G-FETs with a high mechanical stability and devices performance. Through nanopatterning of self-assembled BCP, we fabricate various nanoporous substrates (AES and OES) with excellent regularity and large-area uniformity. While the graphene transferred to FS showed a p-doped property due to contact doping with substrate, graphene transferred to nanopatterned substrates (AES and OES) show intrinsic properties because of partial suspension on the substrate. Considering that graphene on AES and OES also showed a relatively n-doped feature compared to the one transferred to FS, it was found that graphene doping level is severely affected by the geometry of substrates. In addition, G-FETs on OES showed maximum 3.2 folds higher *μ* compared to devices on FS because of the reduced charged impurities and extrinsic scattering. Therefore, we believe that this strategy can contribute to performance enhancements of future electronic and optoelectronic applications based on 2D materials.

## References

[1] Novoselov K.S., Jiang D., Schedin F., Booth T.J., Khotkevich V.V., Morozov S.V., Geim A.K. (2005). Two-dimensional atomic crystals. Proc. Natl. Acad. Sci. USA.

[2] Kim K.S., Zhao Y., Jang H., Lee S.Y., Kim J.M., Kim K.S., Ahn J.-H., Kim P., Choi J.-Y., Hong B.H. (2009). Large-scale pat-tern growth of graphene films for stretchable transparent electrodes. Nature.

[3] Ohno Y., Maehashi K., Yamashiro Y., Matsumoto K. (2009). Electrolyte-Gated Graphene Field-Effect Transistors for Detecting pH and Protein Adsorption. Nano Lett..

[4] Li L., Yu Y., Ye G.J., Ge Q., Ou X., Wu H., Feng D., Chen X.H., Zhang Y. (2014). Black phosphorus field-effect transistors. Nat. Nanotechnol..

[5] Anasori B., Gogotsi Y. (2019). 2D Metal Carbides and Nitrides (MXenes).

[6] Park S.J., Kwon O.S., Lee S.H., Song H.S., Park T.H., Jang J. (2012). Ultrasensitive Flexible Graphene Based Field-Effect Tran-sistor (FET)-Type Bioelectronic Nose. Nano Lett..

[7] He Q., Zeng Z., Yin Z., Li H., Wu S., Huang X., Zhang H. (2012). Fabrication of Flexible MoS_2_ Thin-Film Transistor Arrays for Practical Gas-Sensing Applications. Small.

[8] Georgiou T., Jalil R., Belle B.D., Britnell L., Gorbachev R.V., Morozov S.V., Kim Y.-J., Gholinia A., Haigh S.J., Makarovsky O. (2013). Vertical field-effect transistor based on graphene–WS2 heterostructures for flexible and transparent electronics. Nat. Nanotechnol..

[9] Lee C.H., Kim D.R., Zheng X. (2011). Fabrication of Nanowire Electronics on Nonconventional Substrates by Water-Assisted Transfer Printing Method. Nano Lett..

[10] Varchon F., Feng R., Hass J., Li X., Nguyen B.N., Naud C., Mallet P., Veuillen J.-Y., Berger C., Conrad E.H. (2007). Electronic Structure of Epitaxial Graphene Layers on SiC: Effect of the Substrate. Phys. Rev. Lett..

[11] Wang Y.Y., Ni Z.H., Yu T., Shen Z.X., Wang H.M., Wu Y.H., Chen W., Shen Wee A.T. (2008). Raman Studies of Monolayer Graphene: The Substrate Effect. J. Phys. Chem. C.

[12] Dean C.R., Young A.F., Meric I., Lee C., Wang L., Sorgenfrei S., Watanabe K., Taniguchi T., Kim P., Shepard K.L. (2010). Boron nitride substrates for high-quality graphene electronics. Nat. Nanotechnol..

[13] Li Q., Lee C., Carpick R.W., Hone J. (2010). Substrate effect on thickness-dependent friction on graphene. Phys. Status Solidi.

[14] Chen J.-H., Jang C., Adam S., Fuhrer M.S., Williams E.D., Ishigami M. (2008). Charged-impurity scattering in graphene. Nat. Phys..

[15] Meyer J.C., Geim A.K., Katsnelson M.I., Novoselov K.S., Booth T.J., Roth S. (2007). The structure of suspended graphene sheets. Nat. Cell Biol..

[16] Bolotin K.I., Sikes K.J., Jiang Z., Klima M., Fudenberg G., Hone J., Kim P., Stormer H.L. (2008). Ultrahigh electron mobility in suspended graphene. Solid State Commun..

[17] Du X., Skachko I., Barker A., Andrei E.Y. (2008). Approaching ballistic transport in suspended graphene. Nat. Nanotechnol..

[18] Lloyd D., Liu X., Christopher J.W., Cantley L., Wadehra A., Kim B.L., Goldberg B.B., Swan A.K., Bunch J.S. (2016). Band Gap Engineering with Ultralarge Biaxial Strains in Suspended Monolayer MoS_2_. Nano Lett..

[19] Thiruraman J.P., Fujisawa K., Danda G., Das P.M., Zhang T., Bolotsky A., Perea-Lòpez N., Nicolaï A., Senet P., Terrones M. (2018). Angstrom-Size Defect Creation and Ionic Transport through Pores in Single-Layer MoS2. Nano Lett..

[20] Neri I., López-Suárez M. (2018). Electronic transport modulation on suspended few-layer MoS2 under strain. Phys. Rev. B.

[21] Hu Y., Kumar P., Xuan Y., Deng B., Qi M., Cheng G.J. (2016). Controlled and Stabilized Light-Matter Interaction in Graphene: Plasmonic Film with Large-Scale 10-nm Lithography. Adv. Opt. Mater..

[22] Yim S., Han H.J., Jeon J., Jeon K., Sim D.M., Jung Y.S. (2018). Nanopatterned High-Frequency Supporting Structures Stably Eliminate Substrate Effects Imposed on Two-Dimensional Semiconductors. Nano Lett..

[23] Kanidi M., Dagkli A., Kelaidis N., Palles D., Aminalragia-Giamini S., Marquez-Velasco J., Colli A., Dimoulas A., Li-dorikis E., Kandyla M. (2019). Surface-Enhanced Raman Spectroscopy of Graphene Integrated in Plasmonic Silicon Plat-forms with Three-Dimensional Nanotopography. J. Phys. Chem. C.

[24] Chaste J., Hnid I., Khalil L., Si C., Durnez A., Lafosse X., Zhao M.-Q., Johnson A.T.C., Zhang S., Bang J. (2020). Phase Transition in a Memristive Suspended MoS2 Monolayer Probed by Opto- and Electro-Mechanics. ACS Nano.

[25] Kaushik N., Ghosh S., Lodha S. (2018). Low-Frequency Noise in Supported and Suspended MoS2 Transistors. IEEE Trans. Electron Devices.

[26] Kim B.H., Park S.J., Jin H.M., Kim J.Y., Son S.-W., Kim M.-H., Koo C.M., Shin J., Kim J.U., Kim S.O. (2015). Anomalous Rapid Defect Annihilation in Self-Assembled Nanopatterns by Defect Melting. Nano Lett..

[27] Kim S.O., Solak H.H., Stoykovich M.P., Ferrier N.J., de Pablo J.J., Nealey P.F. (2003). Epitaxial self-assembly of block copoly-mers on lithographically defined nanopatterned substrates. Nature.

[28] Kim B.H., Kim J.Y., Kim S.O. (2013). Directed self-assembly of block copolymers for universal nanopatterning. Soft Matter.

[29] Kim B.H., Kim J.Y., Jeong S.-J., Hwang J.O., Lee D.H., Shin D.O., Choi S.-Y., Kim S.O. (2010). Surface Energy Modification by Spin-Cast, Large-Area Graphene Film for Block Copolymer Lithography. ACS Nano.

[30] Novoselov K.S., Geim A.K., Morozov S.V., Jiang D., Zhang Y., Dubonos S.V., Grigorieva I.V., Firsov A.A. (2004). Electric Field Effect in Atomically Thin Carbon Films. Science.

[31] Kim B.H., Byeon K.-J., Kim J.Y., Kim J., Jin H.M., Cho J.-Y., Jeong S.-J., Shin J., Lee H., Kim S.O. (2014). Negative-Tone Block Copolymer Lithography by In Situ Surface Chemical Modification. Small.

[32] Kim B.H., Choi Y., Kim J.Y., Shin H., Kim S., Son S.-W., Kim S.O., Kim P. (2014). Wrinkle-Directed Self-Assembly of Block Copolymers for Aligning of Nanowire Arrays. Adv. Mater..

[33] Kim J.Y., Kim H., Kim B.H., Chang T., Lim J., Jin H.M., Mun J.H., Choi Y.J., Chung K., Shin J. (2016). Highly tunable refractive index visible-light metasurface from block copolymer self-assembly. Nat. Commun..

[34] Kim B.H., Koo C.M., Shin D.O., Jeong S.J., Kim S.O. (2007). The Synthesis of Random Brush for Nanostructure of Block Co-polymer. Macromol. Symp..

[35] Kim B.H., Lee H.M., Lee J.-H., Son S.-W., Jeong S.-J., Lee S., Lee D.I., Kwak S.U., Jeong H., Shin H. (2009). Spontaneous Lamellar Alignment in Thickness-Modulated Block Copolymer Films. Adv. Funct. Mater..

[36] Gu X., Gunkel I., Russell T.P. (2013). Pattern transfer using block copolymers. Philos. Trans. R. Soc. A Math. Phys. Eng. Sci..

[37] Kim B.H., Koo C.M., Jeong S.-J., Shin D.O., Kim S.O. (2007). Self-Assembled Nanostructures of Block Copolymers on Random Copolymer Brush. Solid State Phenom..

[38] Kim B.H., Lee D.H., Kim J.Y., Shin D.O., Jeong H.Y., Hong S., Yun J.M., Koo C.M., Lee H., Kim S.O. (2011). Mussel-Inspired Block Copolymer Lithography for Low Surface Energy Materials of Teflon, Graphene, and Gold. Adv. Mater..

[39] Oh S.J., Uswachoke C., Zhao T., Choi J.-H., Diroll B.T., Murray C.B., Kagan C.R. (2015). Selective p- and n-Doping of Col-loidal PbSe Nanowires To Construct Electronic and Optoelectronic Devices. ACS Nano.

[40] Das A., Pisana S., Chakraborty B., Piscanec S., Saha S.K., Waghmare U.V., Novoselov K.S., Krishnamurthy H.R., Geim A.K., Ferrari A.C. (2008). Monitoring dopants by Raman scattering in an electrochemically top-gated graphene transistor. Nat. Nanotechnol..

[41] Lin Y., Chiu H., Jenkins K.A., Farmer D.B., Avouris P., Valdes-Garcia A. (2009). Dual-Gate Graphene FETs With *f_T_* of 50 GHz. IEEE Electron Device Lett..

[42] Chen J.-H., Jang C., Xiao S., Ishigami M., Fuhrer M.S. (2008). Intrinsic and extrinsic performance limits of graphene devices on SiO_2_. Nat. Nanotechnol..

[43] Kang Y.-J., Kang J., Chang K.J. (2008). Electronic structure of graphene and doping effect on SiO_2_. Phys. Rev. B.

[44] Kim J.Y., Kim J., Kang S.H., Shin D.O., Lee M.J., Oh J., Lee Y.-G., Kim K.M. (2020). Efficient cell design and fabrication of concentration-gradient composite electrodes for high-power and high-energy-density all-solid-state batteries. ETRI J..

